# Delayed Nephron Sparing Surgery for Grade IV Renal Injury

**DOI:** 10.1155/2013/482320

**Published:** 2013-05-15

**Authors:** Parth K. Shah, Ryan W. Frieben, Rowena A. DeSouza

**Affiliations:** Department of Urology, University of Texas Medical School at Houston, 6431 Fannin Street, Houston, TX 77030, USA

## Abstract

Renal injuries are a common occurrence in many trauma cases. The management of these cases varies, but, currently, a conservative, nonoperative approach is the norm. In cases where an operative intervention may be necessary, emergency total nephrectomies are the most commonly performed procedure reported in the literature. There is a dearth in the reporting of other surgical approaches, especially the delayed nephron sparing approach. Here, we present a unique case of a 29-year-old male that underwent a delayed nephron sparing nephrectomy for a persistent urinoma despite appropriate noninvasive therapy.

## 1. Introduction 

Traumatic injuries to the kidney occur in approximately 10% of all trauma cases and occur more often in cases involving blunt trauma [1]. They can be isolated or associated with other intra-abdominal injuries. These associated injuries along with hemodynamic stability and radiographic severity of renal injury determine optimal management strategy [2]. Conservative, nonoperative approach has become widely accepted over the last 2 decades especially in cases of low grade (I-III) renal injuries. In cases of grade V injuries, surgical management, often with nephrectomy, is the norm. Management of grade IV renal injuries is controversial and has changed as new evidence highlights no statistical difference in complication rates between patients managed nonoperatively versus operatively [3]. Nevertheless, in certain settings, surgery is unavoidable and the decision to perform nephron sparing (partial) nephrectomy versus complete nephrectomy rests on location of injury and associated vascular damage [4]. The literature review demonstrates that in cases where surgery is necessary, emergency nephrectomies are performed more commonly than nephron sparing surgery. The occurrence of delayed nephrectomy, specifically delayed partial nephrectomy, is not known. Therefore, this case involving the successful management of an isolated grade IV injury in a 29-year-old male with delayed nephron sparing surgery is a notable event. 

## 2. Case Report

A 29-year-old otherwise healthy Hispanic male presented to the emergency department one day after falling 12 feet from a ladder and onto a fence while at work. He complained of nausea, left flank pain, and hematuria. The patient had a brief loss of consciousness after the fall but denied persistent headaches, dizziness, sensory changes, confusion, or vomiting. 

**Figure 1:**
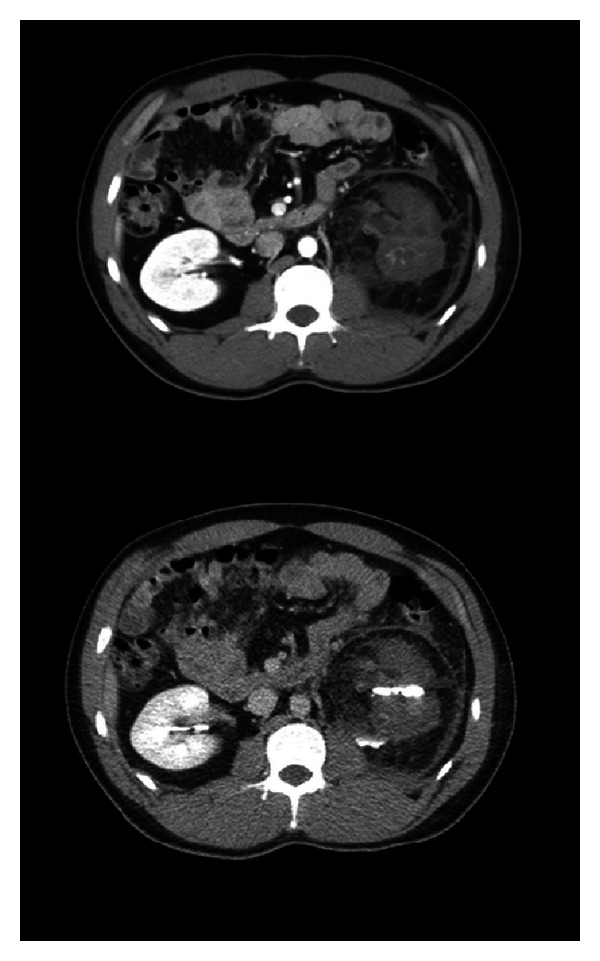
CT abdomen and pelvis with contrast upon presentation to ED showing Grade IV renal laceration.

Initially in the ED, the patient was normotensive (128/86 mm/Hg), tachycardic (132 bpm), tachypneic (20 RR) and febrile (100.6 F). A focused assessment with sonogram for trauma (FAST) exam was performed and did not show any occult bleeding. A contrast CT of the chest, abdomen, and pelvis revealed a grade IV left renal injury with a thrombus seen in the left inferior renal artery causing devascularization of the lower pole. There was also a small, nonocclusive main renal vein thrombus as well as a small amount of extravasation from the lower pole renal pelvis into the perirenal space and a small retroperitoneal hematoma. The right kidney and other organs appeared normal (Figure 1). Initial hemoglobin was 12.3 and creatinine was 1.5. The physical exam was only significant for mild left flank tenderness to palpation. After administration of pain medication, the tachycardia resolved. At this time a conservative approach was deemed and the patient was scheduled to be reimaged in 48 hours to monitor the outcome of the urinary extravasation.

The patient was taken to the OR the next day and underwent a cystoscopy and left retrograde pyelogram that showed contrast extravasation at the inferior pole calyx. A left-sided 6 Fr × 26 cm JJ stent was placed along with a 16 Fr foley catheter. Two days later, a repeat contrast CT scan of the abdomen and pelvis was performed due to fever and a rising WBC count. The repeat scan revealed an interval increase in size and complexity of the posterior pararenal space fluid collection, compatible with a urinoma with active urinary contrast extravasation from the left renal pelvis and left lower pole renal calyces. However, since the ureteral stent was in proper placement and there was no evidence of hydronephrosis or infection, IR determined that a percutaneous nephrostomy (PCN) was not necessary. The patient was subsequently discharged home and instructed to follow up in clinic in 5-6 weeks for evaluation and ureteral stent removal. 

**Figure 2:**
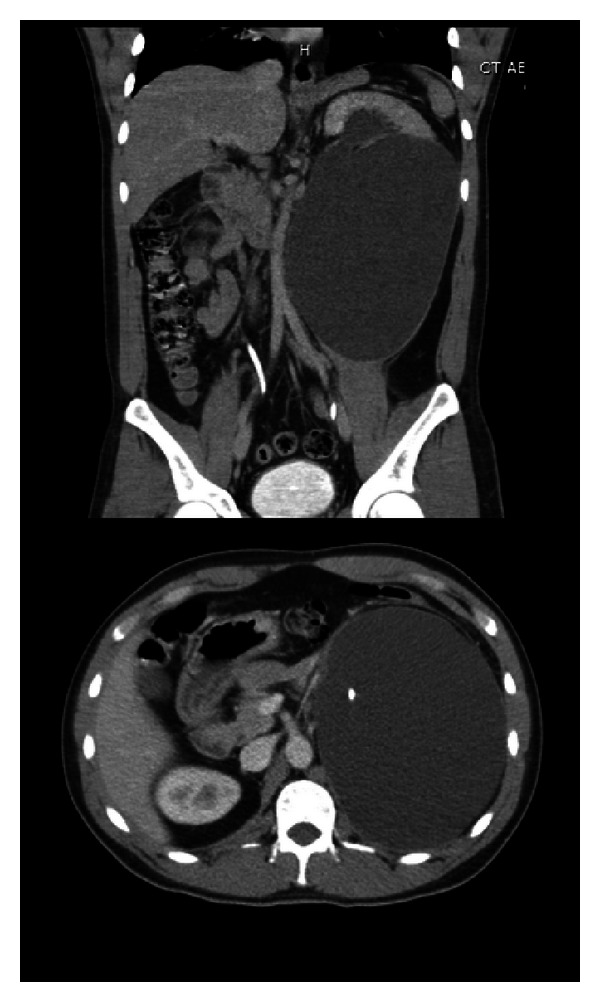
Urinoma seen on repeat CT abdomen and pelvis with contrast after placement of ureteral stent.

The patient reported no symptoms during the intervening period. An interval contrast CT abdomen and pelvis scan showed a large left renal infrapelvic urinoma with distention of the left proximal collecting system despite the presence of an intact appearing left ureteral stent. Devascularization of the left kidney inferior pole was also noted (Figure 2). The patient was immediately admitted and underwent CT-guided placement of an 8.5 Fr drainage catheter into the urinoma. He was discharged home with the drain in place and instructed to follow up in clinic in 2 weeks.

**Figure 3:**
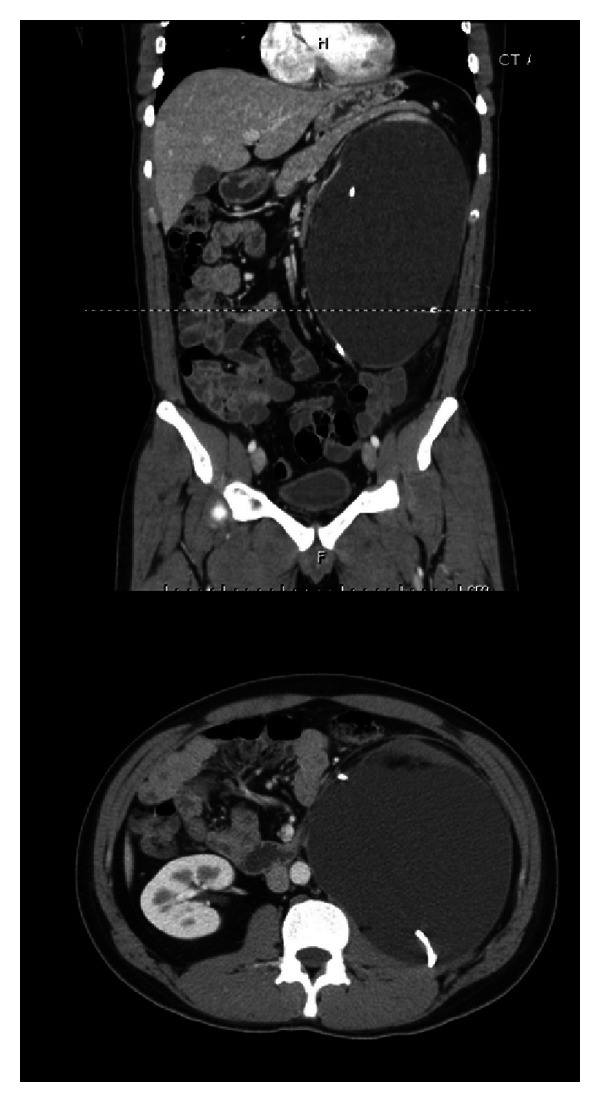
Persistent urinoma seen on repeat CT abdomen and pelvis with contrast after placement of percutaneous nephrostomy tube and ureteral stent.

On follow-up visit, the patient was once again asymptomatic. Imaging showed a slight interval increase in size of the left-sided subcapsular urinoma despite placement of a pigtail drainage catheter within the fluid collection. The urinoma demonstrated a mass effect upon the pancreas, spleen, descending colon, right kidney, and left psoas muscle. The presence of left mild hydronephrosis was unchanged (Figure 3). A retroperitoneal exploration for possible repair of renal laceration or possible nephrectomy was scheduled.

**Figure 4:**
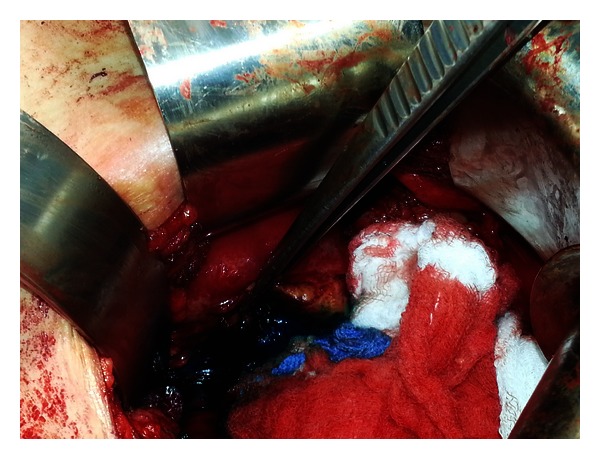
Methylene blue seen extravasating through the site of detachment of the lower pole.

During the procedure it was noted that the lower pole was completely detached from the remainder of the kidney. Consequently, portions of nonviable tissue were removed. There was no evidence of infection; however, moderate amount of fibrotic changes, most likely secondary to his history of urinary leakage, was present. Fortunately, the renal pelvis was still in continuity with a viable upper midpole. The upper midpole was carefully dissected down to the level of the capsule. The renal pelvis was then injected with methylene blue which was seen extravasating through the defect where lower pole was previously attached (Figure 4). 

**Figure 5:**
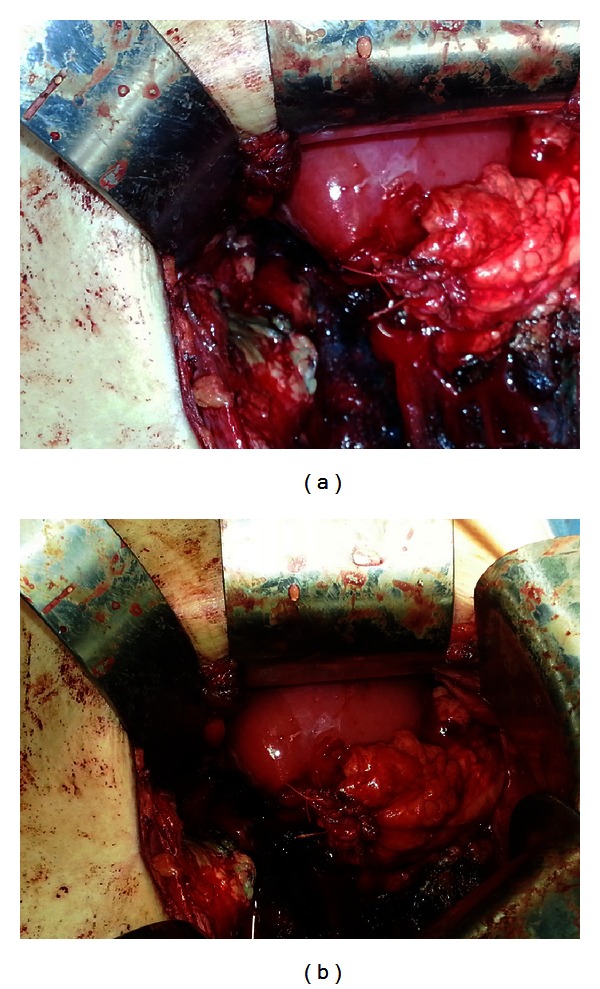
Closing of defects secondary to lower pole detachment and securing closure with Gerota's fascia.

The defects were closed using 4-0 PDS in a running fashion which was performed twice over both defects. Then, an imbricating stitch was performed, closing the renal parenchyma over this defect. The capsule was closed using 1-0 Vicryl in an interrupted horizontal mattress fashion. Gerota's fascia was secured over this defect. At this point, there was no evidence of leakage and hemostasis was achieved (Figures 5(a) and 5(b)). A drain was left around the repair and multiple layer closure performed. 

Postop care was complicated by leukocytosis, tachycardia and fever on postop day 2. Blood and urine cultures were negative and chest X-ray revealed atelectasis but no signs of pneumonia were noted. The patient's leukocytosis and fever subsequently resolved on antibiotics and patient was discharged on postop day 4. 

## 3. Discussion

Trauma involving the genitourinary tract accounts for approximately 10% of all trauma cases. 60%–90% of these cases are secondary to blunt trauma. In one retrospective study of 3,580 cases of renal trauma, 3,115 (87%) were due to blunt trauma [1]. Though the mechanism of injury, associated injuries, hemodynamic stability, and severity of the injury all play a role in determining management, it is usually injury severity as based on CT findings that primarily dictates the optimal approach to management [2].

**Figure 6:**
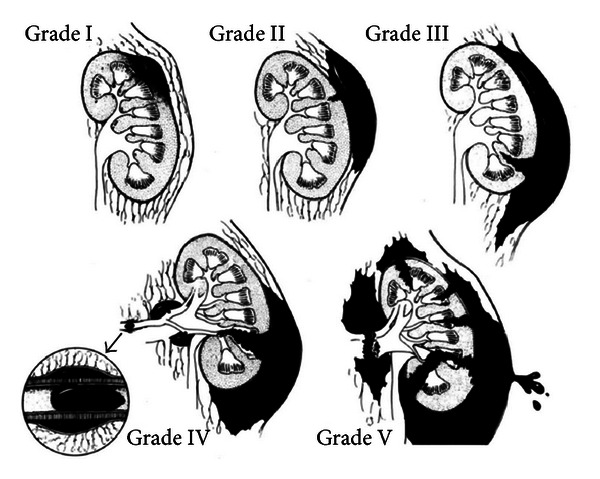
American Association for the Surgery of Trauma (AAST) Organ Injury Severity Score for the kidney as described by Moore et al. (figure derived from Santucci et al. [5]).

The system used to classify renal trauma was created by the Organ Injury Scaling Committee of the American Association for the Surgery of Trauma in 1989. The system grades renal injuries from I to V and bases severity on anatomical disruption (Figure 6; Table 1). Traditionally, grade I to grade III injuries have been successfully managed nonoperatively while grade V injuries usually always undergo surgical exploration and repair.

Management of grade IV injuries is much more controversial as they can be observed and undergo urgent repair or delayed repair. In a study done at San Francisco General Hospital in 2001, grade IV injuries were surgically treated 78% of time with either renorrhaphy (69%) or nephrectomy (9%). In that study, absolute indications for surgical management as deemed by the surgeons included hemodynamic instability; expanding, pulsatile, or uncontained retroperitoneal hematoma; and suspected renal pedicle avulsion. Relative indications included persistent renal bleeding, extracapsular urinary extravasation, nonviable renal tissue, and incomplete staging [5]. This aggressive approach to grade IV injuries has changed as emerging data have shown good results with conservative management. A recent study done at Parkland Hospital in Dallas showed no statistical difference in the complication rates of patients with grade IV renal injury that underwent conservative management versus surgical exploration. Complications of nonoperative management which may require delayed management include delayed hemorrhage, infected urinoma, and, as in our case, urinary extravasation and persistent urinoma [3]. Of these, persistent extravasation or urinoma is the most frequent and occurs in approximately 10%–20% of cases [6].

The management of persistent urinoma is via placement of ureteric stent. Percutaneous placement of drainage tubes and delayed repair can also be attempted if less invasive procedures do not yield satisfactory results [7]. Three studies performed prior to the advent of CT staging evaluated nonoperative management of blunt renal trauma with urinary extravasation. The number of cases that required surgical exploration for persistent extravasation varied from 14% to 20%. In a more recent study done in 1993, only 6% of patients with blunt renal trauma needed to undergo surgical treatment for persistent urinoma despite stenting. The authors of that study concluded that even though urinary extravasation often is self-sealing, it may require more aggressive management if it is due to renal trauma resulting in devitalized segments, such as in this case [8]. However, aggressive management is rare as demonstrated by a study published in 2012 in which only 2 out of 72 (3%) patients with high grade renal injuries with urinary extravasation failed stent management and had a subsequent renorrhapy [7]. 

Once a surgical approach is deemed necessary, then the location of injury (i.e., if it is polar or interpolar) or associated vascular damage determines if nephron sparing surgery is a viable option. It seems that nephrectomies are the preferred management option as partial nephrectomies are rarely, if ever, performed in high grade renal trauma cases. In one study, only 14% of (2/7) cases of Grade IV renal trauma that were managed surgically underwent partial nephrectomy whereas 4 (57%) underwent a nephrectomy [4]. In another study, 5/26 (19%) high grade renal injuries secondary to blunt trauma were managed by nephrectomy—none by partial nephrectomy. In the same study, 1/25 (4%) of high grade renal injuries secondary to penetrating trauma were managed by partial nephrectomy, whereas 8/25 (32%) were managed with nephrectomy [9]. In a study of penetrating injuries, 6/49 (12%) high grade injuries were managed with partial nephrectomy while 13/49 (26%) were managed with nephrectomy [10].

There have been multiple studies that investigated the management of high grade renal injuries in which a partial nephrectomy was not performed at all and surgical management was either with a renorrhaphy or a nephrectomy [2, 3]. The only study where partial nephrectomies were performed more often than nephrectomies (24% versus 10%) was performed by Santucci and McAninch in 2001. However, the data were presented in a manner in which it is difficult to gauge exactly what type of repair was done in each case as some injuries required more than one type of repair [5]. Furthermore, though partial nephrectomies have been performed, it is unclear how many of them are done in a delayed fashion as the timing of the surgery is not commonly reported in the literature. Therefore, the true incidence of delayed nephron sparing surgery is unknown and warrants more inquiry. 

From the previous literature review, it can be appreciated that as standards of care and imaging techniques have improved over the past 2 decades, the incidence of surgical repair of renal injuries has subsequently decreased and is now becoming rare. This is highlighted by the lack of reporting of the incidence of delayed partial nephrectomy in the literature. Thus, the occurrence of a delayed partial nephrectomy secondary to persistent urinoma is rare and this makes the utilization of such an approach a notable event.

## Figures and Tables

**Table 1:** AAST Organ Injury Severity Score for the kidney (table derived from Santucci and McAninch [5]).

Grade^a^	Type	Description
I	Contusion	Microscopic or gross hematuria, urologic studies normal

II	Hematoma	Subcapsular, nonexpanding without parenchymal laceration
	Hematoma	Nonexpanding perirenal hematoma confirmed to renal retroperitoneum
	Laceration	Parenchymal depth of renal cortex <1.0 cm without urinary extravasation

III	Laceration	Parenchymal depth of renal cortex <1.0 cm without collecting system rupture or urinary extravasation

IV	Laceration	Parenchymal laceration extending through renal cortex, medulla, and collecting system
	Vascular	Main renal artery or vein injury with contained hemorrhage

V	Laceration	Completely shattered kidney
	Vascular	Avulsion of renal hilum that devascularizes kidney

^a^Advanced one grade for bilateral injuries up to Grade III.

## References

[1] Buckley JC, McAninch JW (2011). Revision of current american association for the surgery of trauma renal injury grading system. *Journal of Trauma*.

[2] Buckley JC, McAninch JW (2006). Selective management of isolated and nonisolated grade IV renal injuries. *Journal of Urology*.

[3] Shariat SF, Jenkins A, Roehrborn CG, Karam JA, Stage KH, Karakiewicz PI (2008). Features and outcomes of patients with grade IV renal injury. *The British Journal of Urology International*.

[4] Aragona F, Pepe P, Patane D, Malfa P, D’Arrigo L, Pennisi M (2012). Management of severe blunt trauma in adult patients: a 10 year retrospective review from an emergency hospital. *The British Journal of Urology International*.

[5] Santucci RA, McAninch JM (2001). Grade IV renal injuries: evaluation, treatment, and outcome. *World Journal of Surgery*.

[6] Moudouni SM, Patard JJ, Manunta A, Guiraud P, Guille F, Lobel B (2001). A conservative approach to major blunt renal lacerations with urinary extravasation and devitalized renal segments. *The British Journal of Urology International*.

[7] Long JA, Fuard G, Descotes JL (2013). High-grade renal injury: non-operative management of urinary extravasation and prediction of long-term outcomes. *The British Journal of Urology International*.

[8] Matthews LA, Smith EM, Spirnak JP (1997). Nonoperative treatment of major blunt renal lacerations with urinary extravasation. *Journal of Urology*.

[9] Davis KA, Reed RL, Santaniello J (2006). Predictors of the need for nephrectomy after renal trauma. *Journal of Trauma*.

[10] Nicol AJ, Theunissen D (2002). Renal salvage in penetrating kidney injuries: a prospective analysis. *Journal of Trauma*.

